# Heart Failure Care: Testing Dyadic Dynamics Using the Actor-Partner Interdependence Model (APIM)—A Scoping Review

**DOI:** 10.3390/ijerph19041919

**Published:** 2022-02-09

**Authors:** Izabella Uchmanowicz, Kenneth M. Faulkner, Ercole Vellone, Agnieszka Siennicka, Remigiusz Szczepanowski, Agnieszka Olchowska-Kotala

**Affiliations:** 1 Department of Nursing and Obstetrics, Faculty of Health Sciences, Wroclaw Medical University, ul. K. Bartla 5, 51-618 Wroclaw, Poland; izabella.uchmanowicz@umw.edu.pl; 2 School of Nursing, Stony Brook University, Nicolls Road, Stony Brook, NY 11794, USA; kenneth.faulkner@stonybrook.edu; 3 Department of Biomedicine and Prevention, University of Rome “Tor Vergata”, Via Montpellier 1, 00133 Rome, Italy; ercole.vellone@uniroma2.it; 4 Department of Physiology and Pathophysiology, Wroclaw Medical University, ul. T. Chałubińskiego 10, 50-368 Wroclaw, Poland; agnieszka.siennicka@umw.edu.pl; 5 Department of Computer Science and Systems Engineering, Wroclaw University of Science and Technology, ul. Janiszewskiego 11/17, 50-372 Wroclaw, Poland; remigiusz.szczepanowski@pwr.edu.pl; 6 Department of Humanities and Social Science, Wroclaw Medical University, ul. Mikulicza-Radeckiego 7, 50-368 Wroclaw, Poland

**Keywords:** heart failure, self-care behaviors, Actor–Partner Interdependence Model (APIM), caregiver, dyad, dyadic care, patients

## Abstract

Self-care behaviors are essential for the effective treatment of heart failure (HF), and poor self-care may lead to adverse clinical events in patients with HF. A growing body of literature addresses the need to analyze the characteristics of both patient and caregiver since they are in mutual, long-term interaction, and their reactions to events are dependent on each other. One of the most common approaches for analyzing data on HF self-care dyads is the Actor–Partner Interdependence Model (APIM). The purpose of this study was to conduct a scoping review to answer the following question: what did we learn from HF dyadic studies based on the APIM approach? *Medline, Academic Search Ultimate,* and *CINAHL Complete* databases were searched, using the terms “dyad,” “dyadic,” and “heart failure,” for studies published between 2009 and April 2021. Fifteen studies were reviewed from a pool of 106 papers. Studies using the APIM approach revealed interrelated patient and caregiver characteristics that influence self-care and explain many complex dyadic behaviors. Our analysis provided evidence that (1) APIM is a useful analytical approach; (2) a family-oriented approach can improve the functioning of a patient with HF; and (3) social support from caregivers significantly enhances patients’ adaptation to illness.

## 1. Introduction

Heart failure (HF) is a complex, heterogeneous, increasingly prevalent cardiovascular disorder with high morbidity and mortality [1,2]. Self-care behaviors are essential for the effective treatment of heart failure, and poor self-care may lead to adverse clinical events in patients with HF, including repeated hospitalizations, poor quality of life, and increased mortality [3]. Several factors contribute to adequacy of self-care. Depression [4], sleep disturbances [5], impaired cognition, multiple comorbid conditions [6], and low level of awareness of illness decline all limit self-care [7]. The involvement of the partner, i.e., the informal caregiver who assists the patient with daily self-care, is crucial. Caregiver mental health, strain, and contributions to self-care predict patient clinical events in heart failure [8,9,10]. A caregiver is often someone very close to the patient, such as a spouse or an adult child, who helps the patient with daily functioning and has the potential to influence the trajectory of this chronic disease. Increasingly, researchers are using a dyadic approach to study self-care in HF because they have realized that self-care is a dyadic phenomenon in which patients and their caregivers are an interdependent team working within their life context and that the way they appraise illness as a team influences management of the disorder [11]. HF patients usually have a partner with whom they make day-to-day decisions about symptom management but also about diet and how to deal with worsening symptoms. Dyadic HF research has shown that good relationships with a partner and other people [12,13], knowledge regarding HF of each member of the dyad [14], congruence in symptom assessment, and agreement on who is providing self-care [15] influence HF behavior and may determine a patient’s outcome. Within this dyad, caregivers influence patient self-care and patients influence caregiver’s contribution to self-care. Patients struggle to perform self-care; therefore, the contribution of informal caregivers is fundamental, and a dyadic approach is necessary. Therefore, the dyadic approach to self-care allows a more accurate assessment the factors determining effective self-care in HF by including both the patient and the caregiver [16].

## 2. Theoretical Background

Studies conducted on caregiver participation in self-care in HF and other chronic conditions have shown that taking the caregiver role into account improves patient outcomes. Since HF patients and their caregivers influence each other in self-care, investigators have started to approach self-care studies using dyadic approaches because they allow controlling for the interdependence between patients and caregivers. The theoretical framework for research on dyadic care in HF is a combination of three theories: the Theory of Dyadic Illness Management (TDIM) [11], Situation-Specific Theory of Heart Failure Self-Care (SSTHFSC) [17,18], and the Situation-Specific Theory of Caregiver Contribution to Heart Failure Self-Care (SSTCCHFSC) [19]. The TDIM illustrates that management of disease is a dyadic process and describes the interdependency of the patient and the caregiver. The Situation-Specific Theory of Heart Failure Self-Care illustrates the unique aspects of self-care in patients with HF. The Situation-Specific Theory of Caregiver Contribution to Heart Failure Self-Care describes factors influencing caregiver contribution to HF self-care as well as outcomes of this contribution. Research on dyadic self-care in HF focuses on the joint management of this specific disease by the adult patient and caregiver. Many previous studies of self-care in HF have focused on either the patient [20,21,22,23] or the caregiver [9,24,25]. There are also studies investigating dyads with chronic illnesses, but their focus was more about dyadic appraisal and coping (i.e., spousal involvement and communication between dyadic partners) [26]. A dyadic approach to the care of a patient with HF emphasizes the joint efforts of both members in coping with the disease and the interdependency of the two members of the dyad on effectiveness of HF self-care.

HF is a heterogeneous disease, both in terms of patient health status as well as caregiver experience and tasks. The time required in HF caregiving is highly variable and depends on several factors, including the severity and stability of HF, the presence of comorbidities, impairments to physical and/or cognitive function, the complexity of the treatment regimen, and other situational aspects [27]. During the disease, as the disease advances, the experience of HF becomes characterized by continuous management of progressive and pervasive symptoms (e.g., dyspnea, fatigue, edema, insomnia) that severely compromise the quality of life [18,28]. Additionally, patients with advanced heart failure have an uncertain disease trajectory, and this places a significant burden on heart failure caregivers [29]. Higher levels of comorbid conditions are associated with family caregivers feeling fewer positive feelings about providing care [30], and higher patient with HF functional class (worse symptom severity) is significantly associated with greater caregiver anxiety and general stress [31]. Studies across caregiving contexts suggest that caregivers of patients with more severe illness may need the most support [32,33,34].

## 3. Theory of Dyadic Illness Management

According to the TDIM [11], illness management is a dyadic process involving both the patient and the caregiver. A distinctive feature of the dyadic approach to patient care is the focus on dyadic health (i.e., the health of the dyad rather than the care of either the patient or the caregiver). According to the TDIM, the goal of care is to optimize the health of both members of the dyad. The way both members of the dyad appraise the illness can affect the health of them both. Because this dyad is an interdependent team, the way they appraise illness affects how they engage in management behaviors. In many aspects, congruent appraisals of the disease by both dyadic partners can lead to better collaboration in managing illness and ultimately better health. It is best if the patient and the caregiver have greater congruence in the appraisal of symptoms, care values, and preferences. Appraisal influences disease-management behaviors, which occur on a continuum within the dyad. Only one of the care partners is involved in disease management at the low end of the spectrum, whereas both members of the dyad participate in disease-management behaviors at the high end of the spectrum. The theory posits that higher levels of collaboration between the two members of the dyad are associated with better health [11,35]. Collaboration between dyadic partners should include decision making, symptom management, and general health behaviors of both partners. The TDIM also assumes that dyadic management behaviors are influenced by the state of health of the patient and caregiver, the dyadic typology, and the contextual factors linked with social support or culture.

### 3.1. The Situation-Specific Theory of Heart Failure Self-Care

The Situation-Specific Theory of HF self-care was created based on real-life experiences of caring for patients with HF. This theory was published in 2008 [17] and then updated in 2016 given recent empirical findings [18]. According to this theory, self-care includes three separate and interrelated processes: *maintenance* (adherence to recommendations and healthy behaviors, including taking medication, following low-salt diet, and maintaining physical activity), *symptom perception* (monitoring, detecting, interpreting, and labeling signals from the body), and *management* (a behavioral response to emerging symptoms). All processes require knowledge and skill, but the most demanding process is *management*. The SSTHFSC emphasizes that the “naturalistic decision-making” process occurs in real-life situations and dynamic environments with incomplete information, competing needs, time pressure, and high levels of stress due to the potentially life-threatening nature of the event. Patient decisions in self-care in HF are based on both objective data (e.g., weight gain) and subjective data (e.g., fatigue). The SSTHFSC identifies several factors that influence self-care decisions, including person-related factors (e.g., cultural identify, health literacy, socioeconomic status), problem-specific factors (e.g., co-morbidities, including cognitive impairment), and environmental factors (e.g., lack of social support). Given these factors, self-care decisions made by patients sometimes are inconsistent and even wrong.

### 3.2. The Situation-Specific Theory of Caregiver Contribution to HF Self-Care

The Situation-Specific Theory of Caregiver Contribution to HF Self-Care is based both on the SSTHFSC and the Middle Range Theory of Self-Care of Chronic Illnesses [36] and describe factors at caregiver (e.g., age), patient (e.g., cognition), and dyadic level (e.g., the relationship between the patient and the caregiver) that influence the extent to which caregivers contribute to support HF patient self-care. Similar to the SSTHFSC, caregiver contributions to HF self-care include the dimensions of caregiver contribution to self-care maintenance, symptom monitoring, and perception and self-care management. In addition, the theory includes a mediator, which is the caregiver’s self-efficacy that mediates the relationship between the contributors and caregiver contributions to self-care, and the outcomes, which can be related to patient (e.g., quality of life) and caregiver (e.g., self-esteem).

### 3.3. The Actor–Partner Interdependence Model

**Figure A1:**
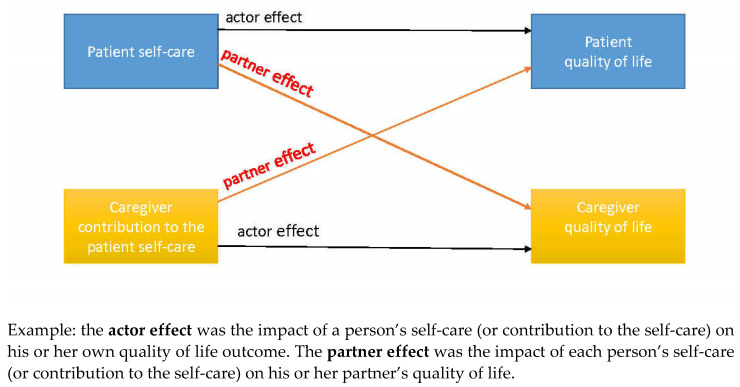
The Actor–Partner Interdependence Model (APIM) used to analyze dyadic data.

The interdependency between the patient and the caregiver needs to be taken into consideration when analyzing data on dyads. The Actor–Partner Interdependence Model (APIM) is the most current methodology for analyzing data on HF self-care dyads. The APIM is based on the Interdependence Theory [37], which assumes that people influence each other’s experiences by interacting with each other. Researchers emphasize the need to analyze the characteristics of both patient and caregiver since they are in a mutual, long-term interaction, and their reactions to events are dependent on each other. The APIM model for HF dyadic care studies is usually supported by paired regression analyses of the relationship within the couple, i.e., a regression method that does not require independent observations (see Appendix A, Figure A1). The APIM model specifies how the independent variable of an individual may impact their own dependent variable (actor effects) as well as the partner’s dependent variable (partner effects) [38]. There are three types of variables in the APIM model: (1) between-dyads variables, (2) within-dyads variables, and (3) mixed variables. Between-dyads variables are those that vary across dyads but are the same for both members of the dyad (e.g., years spent in a relationship). A within-dyads variable varies across the members of the dyad, but each dyad would have the same total score as all other dyads (e.g., if the study consists only of heterosexual couples, this variable will be gender: male and female). A mixed variable is one that has variation both within and between dyads (e.g., age of members). It is possible to estimate actor and partner effects for mixed variables only. Between- and within-dyads variables can be estimated as main effects. Additionally, various interactions can be tested based on the model [39]. An extended model of the APIM, the Actor–Partner Interdependence Mediation Model (APIMeM), allows to assess mediation in dyadic data [40].

To date, there has been little consistency in how data on HF care dyads have been analyzed. As mentioned above, researchers suggest analyzing dyadic data with the APIM model because it takes interactions between dyadic partners into consideration. In recent studies, APIM has been shown to be effective in identifying the determinants of effective dyadic coping with HF. Many authors emphasize the need to continue using this method of analysis [9,28,29]. Since dyadic analysis is becoming a new approach to studying HF self-care, a systematic review of these studies would help orientate future studies on this topic. The purpose of our article was to review the existing evidence on self-care in HF patient/caregiver dyads based on the APIM approach. Establishing the status of existing research in self-care in HF allowed us to systematize recent findings and point out potential areas worthy of further exploration.

## 4. Data Search

Several databases, including *Medline, Academic Search Ultimate,* and *CINAHL Complete,* were searched for relevant literature. Search terms were entered as keywords and included *dyad, dyadic,* and *heart failure*. The search was restricted to 2009–April 2021. Citations of retrieved papers were hand-searched to identify additional relevant studies. Studies were eligible for inclusion if they (1) were published in peer-reviewed journals written in English; (2) used either the APIM or his extended version, the APIMeM, to analyze data; and (3) involved adult population (over 18 years old). The analysis excluded articles focusing on dyadic typologies, the development of methods and research instruments, qualitative tests, longitudinal studies, discussions and conceptualizations about the health of dyads as a unit, and dyadic interventions. We also excluded studies on mixed dyads, including patients with HF and other diseases.

## 5. Results

**Figure 1:**
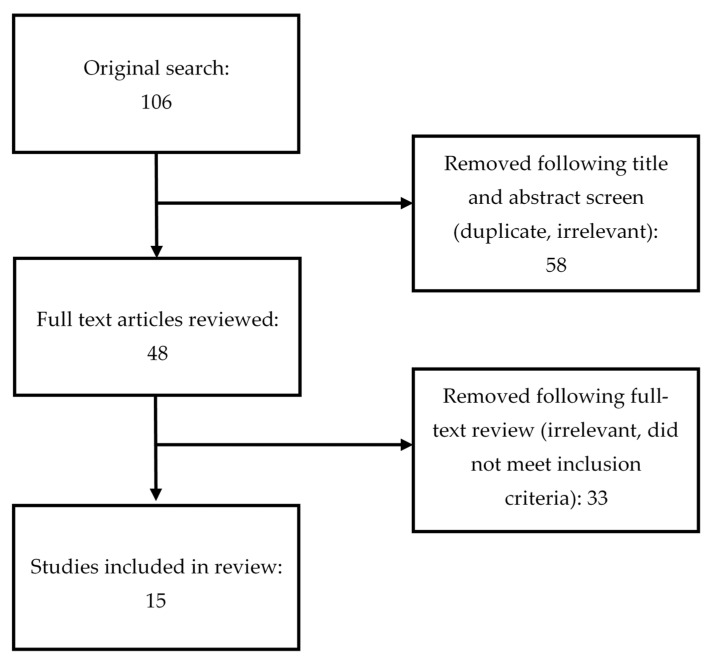
PRISMA table outlining literature search.

The initial search yielded 106 articles. After removal of duplicates, 48 original articles remained. Full text of all resulting papers were reviewed for further assessment, and another 33 were excluded because they did not meet the inclusion criteria, leaving 15 studies for inclusion in this review (see Figure 1). Table 1 provides a detailed description of each study in terms of country, the sample, the aim of the study, main outcome measures, findings, results, and conclusions.

### 5.1. Summary of Findings

#### 5.1.1. Quality of Life and Emotional Aspects of Dealing with HF

Analysis of the dyadic studies demonstrates that managing HF can be very emotional for both the patient and the caregiver [42]. Both partners in the dyad experience high levels of emotional distress [43]; however, patients seem to experience more depressive symptoms than their spouses [41,51]. Greater emotional distress has been associated with poor quality of life. Two studies focused on the caregiver and provided evidence that quality of life of the patient is better if the care partner has no depressive symptoms or anxiety [42,43]. Sleep also is particularly important for caregivers. It has been empirically demonstrated that the presence of sleep disorders significantly affects the mental health of the caregiver [44]. What positively affects the caregiver’s quality of life is the patient’s adherence to therapy [48]. Better emotional well-being of the dyad also is associated with better relationship quality [41]; however, having a good relationship is not “protective” against anxiety and depression for caregivers [53]. If either member of the dyad senses that they are not in control over HF, the emotional well-being of the dyad may become worse [50]. Incongruent collaboration of partners in HF management also affects the emotional well-being of the dyad [51]. Studies on emotional state and quality of life in dyadic caring in HF conclude that interventions are needed to alleviate depressive symptoms in both dyadic partners [42,43]. It appears that nurses can increase dyadic caring by providing the dyadic partners with social support [49].

#### 5.1.2. Dyadic HF Self-Care Confidence

Self-care confidence, or self-care self-efficacy, is the extent to which one feels able to perform regular self-care (patient) and contribute to patient self-care (caregiver) despite difficulties [47]. Research on dyadic self-care has shown that poor self-efficacy affects the ability of the patient and caregiver to engage. Patients with greater self-care confidence are more engaged in self-care behaviors [45]. Studies on dyadic care in HF also show that self-care confidence is not typically equal between the members of the dyad, with caregivers demonstrating greater self-care confidence than patients [16]. However, there has also been heterogeneity in confidence across the dyads, suggesting self-care confidence is adequate in some dyads but is insufficient to support self-care in others [16].

Several factors are associated with better self-care confidence, including female gender, education, social support experienced by both partners, caregiver’s mental health, relationship quality [16], and mutuality within the dyad [45]. Mutuality, defined as the extent to which there is an emotional investment and mutual support in the dyad, was predictive of reduced burden in the caregiver [45]. Therefore, it is best when the caregiver perceives and recognizes mutuality in the relationship with the patient [52].

#### 5.1.3. Maintenance and Management

Data from APIM studies show low levels of daily HF maintenance and management in both partners in HF self-care [46,47]. Not surprisingly, mild cognitive impairment in the patient reduces self-care [46]. Better self-care maintenance is usually achieved in dyads where the caregiver is a woman. Indeed, there is evidence that female gender of the caregiver was a significant predictor of better patient self-care maintenance [46]. This most likely arises from a traditional role of women, who mainly care about family health in many societies. There also is evidence that a caregiver is more likely to contribute to HF self-care management if the caregiver is not the patient’s spouse [46]. Involvement in self-care also is associated with the emotional state of the caregiver, the quality of the relationship between the HF patient and the caregiver [47,52,54], and the caregiver’s knowledge of self-care [47]. Patients are more adherent to recommendations when they have a decreased physical quality of life [48]. It seems, therefore, that a greater problem in engaging patients in self-care may arise in patients with a relatively high physical quality of life. Furthermore, an agreement between dyad partners who are responsible for performing self-care tasks may also be an important issue for self-care. Dyads that were congruent not only reported fewer psychosocial problems [55], but also in these dyads, the caregivers were more engaged in caregiving [47].

## 6. Discussion

In this paper, we reviewed studies using the APIM model to summarize how adult patients and their caregivers interact to perform HF self-care. The APIM is a useful analytical approach to evaluating partner and actor effects in the presence of interdependencies between the dyad partners. Using this model, the studies we reviewed provided evidence of several factors that influence effectiveness of HF self-care in dyads. This review showed which topics and tools have already been used and will help researchers identify new areas that have not yet been explored.

Many studies have focused on the emotional aspects of coping with heart failure and the quality of life of dyadic partners [41,42,43,44,51,53]. The analysis of the studies presented here provides evidence that these factors are crucial in the self-care of patients with HF. Previous studies have also found that effective HF coping is highly dependent on the emotional state of both the patient and the caregiver [29,30,56]. It is exhausting for both of them to deal with the issues of HF daily. The quality of relationships as well as the socio-cultural background are all crucial factors in coping with the disease [35,57,58,59]. Clinicians should evaluate the emotional status of HF patients at each visit, specifically looking for depressive symptoms or anxiety. According to APIM studies, sleep disturbances in caregivers are also worth focusing on and should be treated because they affect the patient’s well-being. Clinicians also should inquire about the quality of the relationship, as strained relationships are less successful with self-care. Studies on dyadic partners and the quality of their relationship have highlighted the impact of social support for self-care of patients with HF. Therefore, in addition to the relationship with the caregiver, clinicians should identify other sources of social support.

Qualities of the caregiver are another factor that contributes to success of the dyad. Care partners who are female and are not the spouse of the patient demonstrate better self-care. Notably, however, cultural differences in caregiving have been identified. Gender and partner roles may vary among cultures and should be considered when developing plans for self-care. Clinicians should speak to both the patient and the family to identify the person best-suited to taking on the role of caregiver. Studies using APIM also have shown the importance of caregiver’s knowledge in the management of HF self-care. After identifying the appropriate caregiver, clinicians should include care partners in self-care education and even tailor education so it corresponds with specific responsibilities of each member of the dyad.

The review revealed that the variables in APIM studies of dyadic care in HF are often the same. The advantage of using the same research measure is the ability to compare studies worldwide and across different groups. The disadvantage of this approach is related to the limited scope of research topics devoted to self-care in HF. For example, we found that relatively little research has been dedicated to evaluating the sense of control over the HF trajectory, which is an essential for understanding the illness, developing coping strategies, and appreciating the importance of appropriate self-care [60].

Our review identified several dyadic studies on self-care maintenance, management, and confidence; however, the concept of symptom perception has not yet been explored. Although research on symptom perception and clinical outcomes has been done, it has not been done in the context of a dyad. Future research should evaluate how symptom perception is influenced by dyad typology and emotional factors that have been found to influence other aspects of self-care. Furthermore, studies should be expanded to investigate the relationship between symptom perception, maintenance, management, and risk perception regarding the probability of desired and undesirable effects of HF self-care in both dyadic partners. There is still a lack of studies on risk perception in terms of the likelihood of desired and undesirable effects of HF therapy and how risk perception affects current behavior in both couples. Both partners’ beliefs about health and health conditions could play a role in determining health-related behaviors. Very often, a person’s perception of the condition influences the health choices more than the condition itself [61,62]. Perceived illness susceptibility, perceived benefits of self-care, perceived barriers to healthy habits, and the seriousness of the condition may be other important factors for dyadic partners’ behavior [63,64], and they seem to be worth exploring in the context of self-care in HF. Lastly, although cognitive dysfunction is common in HF and may influence the effectiveness of the dyad, the association between self-care and cognitive dysfunction within the self-care dyad has not been investigated. Given the high prevalence of cognitive dysfunction in HF, it is unknown how cognitive dysfunction influences dyad typology, the roles of each member of the dyad, and the influence of cognitive dysfunction on self-care effectiveness in the context of the dyad.

Some limitations should be mentioned. HF is a very heterogeneous condition, and each patient has different degrees of dependency. Studies that entered the analysis are not consistent with HF criteria. Since, in the review, we did not consider the degree of independence and type of patient, the generalizability of our results is limited. In addition, studies included in the review look at different variables with different types of dyads in each case, which also suggests that the conclusions based on the research should be interpreted with caution.

This article was limited to analysis of studies based on the APIM and the extended APIM, the Actor–Partner Interdependence Mediation Model (APIMeM). Therefore, studies that evaluated HF self-care in dyads but did not use one of these models were not included. The APIM model appears to be appropriate for analyzing the experiences and decisions in a dyad since it uses an approach that takes into account the lack of independence of the dyadic partners. Nonetheless, so far, researchers have identified more complex associations between self-care and other important variables in the context of a HF self-care dyad. Certainly, the reported APIM findings on the factors associated with adherence, symptom management, dyadic confidence, quality of life, and emotional aspects of coping with HF do not exhaust all the dyadic factors.. We did not include in our review studies on types of HF management and studies on the effectiveness of interventions in HF dyads in our review since they did not match the criteria for inclusion.

**Figure A2:**
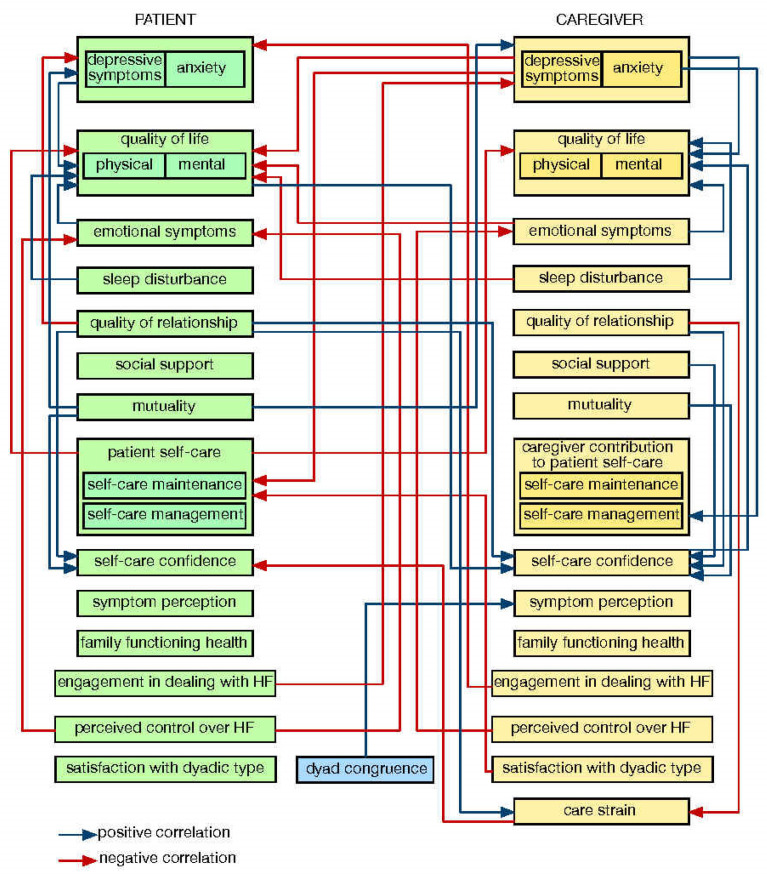
Relationships between caregiver and patient characteristics in studies employing APIM.

**Figure A3:**
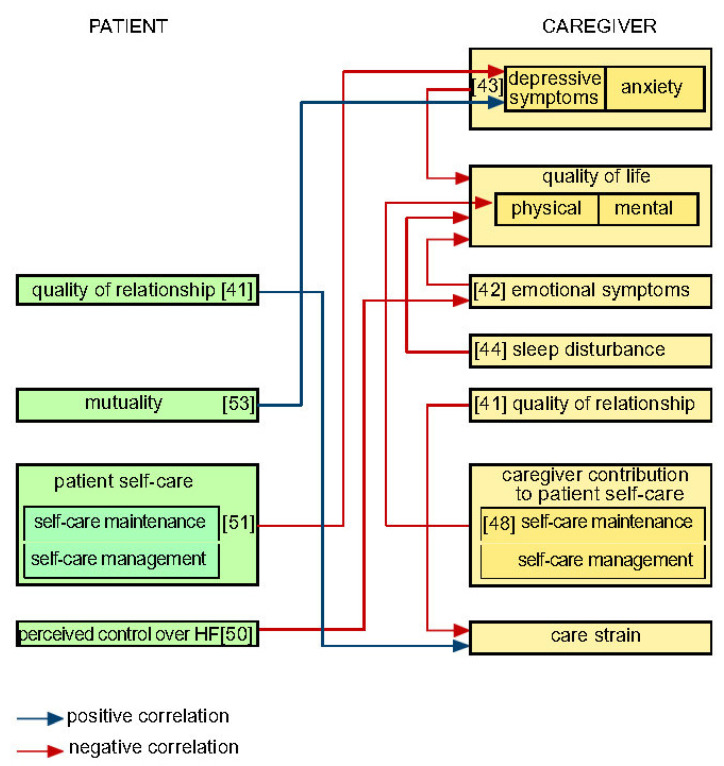
Results regarding caregiver quality of life and emotional aspects of dealing with HF patient.

Considering caregiving factors together with patient factors significantly increases our understanding of patient clinical event risk in HF [8]. The complex interrelationships between patient and caregiver factors revealed by APIM research are depicted in Figure A2 (Appendix A). Figure A2 shows, for instance, that a patient’s self-care outcome (i.e., maintenance, management, symptom perception, or confidence) is significantly related to relationship quality [16,46], dyad congruence [54], as well as caregivers’ depressive symptoms and anxiety [47], mental quality of life [16], satisfaction with dyadic type [54], and patient mutuality [45,52]. Figure A3 (Appendix A), in turn, shows results of APIM research on caregiver quality of life and emotional aspects of dealing with HF patients. Although prior studies indicated similar patterns of relationships [55,56,65,66,67,68], an analysis employing the APIM revealed complex interrelationships between patient and caregiver features. Researchers should consider exploring self-care phenomena using the APIM model to analyze the interdependence between the members of the dyad.

## 7. Conclusions

The frameworks of the Theory of Dyadic Illness Management and the Situation-Specific Theory of Heart Failure Self-Care and Caregiver Contribution to Heart Failure Self-Care are in line with conceptualizing the dyad as a system with interdependence between members of the system. This posited interdependence between the patient and the caregiver has long been used for behavioral treatment (e.g., addictions) and recently became increasingly important for treating chronic diseases [26]. It seems that the APIM model is well suited for dyadic system analysis. Studies using APIM approaches reveal interrelated characteristics of the patient and the caregiver that affect self-care and may explain many complex dyadic behaviors. In this fashion, this approach contributes to a better understanding of the dynamics of the interaction between the caregiver and HF patient. Most of the studies reviewed here conclude that (1) a family-oriented approach can improve the functioning of a patient with heart failure condition, and (2) social support from caregivers significantly enhances patients’ adaptation to illness. Our work’s emerging view is that patient–caregiver interconnections are still enigmatic, and more studies are needed to provide a more complete and coherent picture of self-care in HF. Thus, further research should be carried out to clarify this concept and identify the most critical factors for effective dyadic functioning in HF.

Our review showing the interrelationship of patient and caregiver highlighted the need to include both care partners. It seems necessary not only to continue research showing how the HF patient and caregiver interact but also to simultaneously undertake educational interventions involving members of the dyad as well as nurses and physicians. All caregivers of the HF patient must know and, more importantly, have the aids to facilitate collaboration with the patient. As a preventive measure, some authors have emphasized the importance of physical activity and fitness in HF patients [69,70] as well as the role of nurses in helping HF patients manage their symptoms, particularly through nurse-led educational interventions and assistance with home monitoring through advanced technologies [71,72]. The important role of the social support experienced by the dyad for the effectiveness of its functioning, as revealed in the review, also suggests that some efforts should be made to initiate patient and caregiver support groups in all settings where HF care is provided. In turn, the associations revealed in the review between the psychological status of both dyadic partners and coping with HF suggest that perhaps some patients should receive additional psychological care. Improving the psychological functioning of the dyad could have a positive impact on adherence and help cope with all the challenges posed by the unpredictable trajectory of HF.

## Figures and Tables

**Table 1:** Characteristic of eligible studies.

Author/Year	Country; Sample	Main Outcome Measure(s)	Aim of the Study	Main Results	Main Conclusions
(Lyons et al., 2020) [41]	USA; 60 dyads (patient–spousal caregiver); 67% male patients; M_age_ = 59.5 (patient) and M_age_ = 57.8 (caregiver)	Patient Health Questionnaire (PHQ9); Caregiver Strain Index (MCSI); Emotional-Intimacy-Disruptive-Behavior Scale; Mutuality Scale	To examine the role of interpersonal factors (i.e., concealment and relationship quality) on the depressive symptoms of HF patients and their spouse care partners, care partner strain, and patient hospitalizations	- Patients who conceal their worries and concerns from their care partner may be at risk for increased depressive symptoms and hospitalizations - When patients perceived greater relationship quality with spouse care partners, they reported significantly less depressive symptoms; when spouse care partners perceived greater relationship quality with patients, they reported significantly less care strain - When patients perceived greater relationship quality, spouse care partners reported significantly higher care strain	Patient concealment of worries or concerns (lack of open communication) is a risk factor for patient depressive symptoms and healthcare utilization; one’s own perception of the relationship could have the protective factor.
(Thomson et al., 2020) [42]	UK; 41 dyads (patient–spousal and non-spousal caregivers); 78% male patients; M_age_ = 68.6 (patient) and M_age_ = 65.8 (caregiver)	Brief Symptom Inventory; Minnesota Living with Heart Failure Questionnaire	To examine relationship between emotional symptoms and health-related quality of life	- No differences in emotional symptoms and health-related quality of life between patients with heart failure and their caregivers - Patients’ and caregivers’ emotional symptoms were associated with their own health-related quality of life (actor effect) - Caregivers’ emotional symptoms negatively influenced their partners’ health-related quality of life (partner effect)	Emotional aspects of dealing with heart failure may affect the caregivers as much as their partners who have the illness; the substantial impact of caregivers’ emotional symptoms on the health-related QoL of patients suggest that the caregiver’s emotional well-being needs to be addressed.
(Chung et al., 2009) [43]	USA; 58 dyads (patient–spousal caregiver); 71% male patients; M_age_ = 61.7 (patient) and M_age_ = 57.5 (caregiver)	Brief Symptom Inventory; Minnesota Living with Heart Failure Questionnaire	To explore impact of emotional distress on quality of life (QoL)	- Both patients’ and spousal caregivers’ depressive symptoms and anxiety influenced their own quality of life (actor effect) - Spousal caregivers’ depressive symptoms and anxiety negatively impacted patients’ quality of life, with high depressive symptoms or anxiety in the caregiver spouse predicting poorer quality of life in the patient (partner effect)	Patients with HF may be particularly vulnerable to the emotional distress of their spouse caregivers; interventions to reduce depression and anxiety and to improve patients’ quality of life should include both patients and spouses.
(Al-Rawashdeh et al., 2017) [44]	USA; 78 dyads (patient–spousal caregiver); 56% male patients; M_age_ = 62.2 (patient) and M_age_ = 59.5 (caregiver)	Sleep disturbance: a composite score of four common sleep complaints; Short-Form 12 Health Survey (SF-12)	To determine whether sleep disturbances of patients and their spousal caregivers predicted their own and their partners’ quality of life	- Each individual’s sleep disturbance predicted their own poor physical and mental well-being (actor effect), while spousal caregivers’ sleep disturbance predicted patient’s mental well-being (partner effect)	Patients’ mental well-being is sensitive to their spouses’ sleep disturbance; interventions targeting improving sleep and quality of life may have to include both patients and spousal caregivers.
(Lyons et al., 2015) [16]	Italy; 329 dyads (patient–spousal caregiver or adult children); 56% male patients; M_age_ = 76.8 (patient) and M_age_ = 58.3 (caregiver)	Self-Care of HF Index (SCHFI) and Caregiver Contribution to Self-care of HF Index (CC-SCHFI); Mini-Mental State Examination (MMSE); Minnesota Living with Heart Failure Questionnaire; Short-Form 12 Health Survey (SF-12) (single items); Caregiver Burden Inventory; COPE	To identify individual and dyadic determinants of self-care confidence	- Both patients and caregivers reported moderate levels of confidence, with caregivers reporting slightly higher confidence than patients - Significant heterogeneity in confidence across the dyads - Patient and caregiver levels of confidence were significantly associated with greater patient-reported relationship quality and better caregiver mental health (actor and partner effects) - Patient confidence in self-care was associated with patient female gender, non-spousal care dyads, poor caregiver physical health, and low care strain (partner effect) - Caregiver confidence to contribute to self-care was significantly associated with poor emotional quality of life in patients (partner effect) and greater perceived social support by caregivers (actor effect)	Caregiver mental health must be prioritized; better caregiver mental health and greater relationship quality were the modifiable hallmarks of better self-care confidence in both the patient and the caregiver; the level of confidence in dyads is generallylower-than-adequate.
(Hooker et al., 2018) [45]	USA; 99 dyads (patient–spousal and non-spousal caregiver); 34% male patients; M_age_ = 65.6 (patient) and M_age_ = 57.4 (caregiver)	Mutuality Scale of the Family Caregiving Inventory; Self-care of HF Index (SCHFI) and Caregiver Contribution to Self-care of HF Index (CC-SCHFI); The Zarit Burden Inventory-Short Form (ZBI-SF)	To examine the associations among patient/caregiver self-care confidence and mutuality and caregiver perceived burden.	- Patients and caregivers who perceived better mutuality were more confident in patient self-care (actor effect only) - Caregivers with greater mutuality reported less perceived burden	Mutuality in patient–caregiver dyads is associated with patient self-care and caregiver burden and may be an important intervention target to improve self-care and reduce hospitalizations; there is a need for screening for the quality of the patient–caregiver relationship.
(Bidwell et al., 2015) [46]	Italy; 364 dyads (patient–spousal and non-spousal caregiver); 57% male patients; M_age_ = 76.2 (patient) and M_age_ = 57.4 (caregiver)	Short-Form 12 Health Survey (SF-12); Minnesota Living with Heart Failure Questionnaire; The Barthel Index; Mini-Mental State Examination (MMSE); Caregiver Burden Inventory (CBI); Carers of Older People in Europe Index (COPE); Self-care of HF Index (SCHFI) and Caregiver Contribution to Self-care of HF Index (CC-SCHFI); perceived quality of the relationship between patient and caregiver	To identify determinants of patient and caregiver contributions to HF self-care maintenance (daily adherence and symptom monitoring) and management (appropriate recognition and response to symptoms)	- Both patients and caregivers reported low levels of HF maintenance and management behaviors - Non-spousal relationship type was a significant determinant of higher caregiver contributions to patient self-care management - Better relationship quality was associated with better patient self-care and caregiver contributions to patient self-care although it was the individual’s own perception of the quality of the relationship that was important - Even mild cognitive impairment can have a substantial impact on patient’s self-care	There is the need to examine HF self-care maintenance and management in the context of the patient-caregiver dyad; significant individual and dyadic determinants of self-care maintenance and self-care management included gender, quality of life, comorbid burden, impaired ADLs, cognition, and hospitalizations.
(Buck et al., 2015) [47]	USA; 40 dyads (patient–spousal and non-spousal caregiver); 70% male patients; M_age_ = 71.2 (patient) and M_age_ = 58.8 (caregiver)	Patient Health Questionnaire (PHQ-9); Brief Symptom Inventory; Dyadic Adjustment Scale; Self-care of HF Index (SCHFI) and Caregiver Contribution to Self-care of HF Index (CC-SCHFI)	To describe the dyadic characteristics of mood and perception of the relationship in HF patients and caregivers	- Higher levels of depression or anxiety for the caregiver predicted lower HF self-care maintenance scores for the patient (partner effect) - Higher caregiver anxiety predicted lower caregiver HF self-care management scores, and higher caregiver ratings of relationship quality predicted greater caregiver ratings of self-efficacy (actor effects)	Caregivers’ mood states and perception of the relationship impacts the patient and their own engagement in HF self-care as well as the caregiver’s self-efficacy.
(Vellone et al., 2014) [48]	Italy; 138 dyads (patient–spousal caregiver); 64% male patients; M_age_ = 73.6 (patient) and M_age_ = 70.4 (caregiver)	Self-care of HF Index (SCHFI) and Caregiver Contribution to Self-care of HF Index (CC-SCHFI); Short Form 12 (SF-12)	To explore relationship between self-care behavior and quality of life	- Higher self-care was related to lower physical QoL in patients and caregivers - Higher self-care maintenance in patients was associated with better mental QoL in caregivers - In caregivers, confidence in the ability to support patients in self-care was associated with improved caregivers’ mental QoL	In caregivers, confidence in the ability to support patients in the performance of self-care improved caregivers’ mental QoL; interventions that build the caregivers’ confidence are needed.
(Shamali et al., 2019) [49]	Denmark; 312 dyads (patient–spousal and non-spousal caregiver); 71% male patients; M_age_ = 64.7 (patient) and M_age_ = 58.9 (caregiver)	The Family Functioning, Health and Social Support(FAFHES)	To examine whether perceived social support from nurses is associated with better family functioning of patients with heart failure and their nearest relatives	- The higher the level of family health of the nearest relative, the better the family functioning of the patient (partner effect) - High level of perceived social support from nurses was associated with a higher level of family health and better family functioning in patients with HF and their partner - family health partially (in the patient) and completely (in the nearest relative) mediated the association between social support and family functioning	Social support from nurses could increase the level of family health and family functioning.
(Strömberg et al., 2021) [50]	Sweden; 155 dyads (patient–spousal caregiver); 75% male patients; M_age_ = 71 (patient) and M_age_ = 69 (caregiver)	Control Attitude Scale; Beck Depression Inventory; Short-Form 36	To examine on whether the patients’ perceived control over the management of HF and depressive symptoms predicts their own and their spouses’ physical and emotional well-being and depressive symptoms	- Perceived control over HF was significantly associated with their partners’ emotional well-being - Perceived control over HF had actor effect on emotional well-being for patients	Lack of control over heart disease in any member of the dyads makes their partner feel more insecure and worried; perceived control should be routinely assessed in both patients and spouses during HF follow-up.
(Lyons et al., 2018) [51]	USA; 60 dyads (patient–spousal caregiver); 67% male patients; M_age_ = 59.4 (patient) and M_age_ = 57.7 (caregiver)	European Heart Failure Self-Care Behavior Scale (EHFScB-9); Self-care of HF Index (SCHFI) and Caregiver Contribution to Self-care of HF Index (CC-SCHFI); Patient Health Questionnaire (PHQ9)	To examine the role of congruent engagement in HF-management behaviors on the depressive symptoms of the couple living with HF	- Higher levels of engagement by one’s partner were associated with lower levels of depressive symptoms for both membersof the couple - When couples engage in similar levels of HF-management behaviors, spouses experience lower depressive symptoms	Partner’s level of engagement plays an important role in managing the illness
(Vellone et al., 2018) [52]	Italy; 366 dyads (patient–spousal and non-spousal caregiver); 56% male patients; M_age_ = 71.9 (patient) and M_age_ = 58.6 (caregiver)	Mutuality Scale; Self-care of HF Index (SCHFI) and Caregiver Contribution to Self-care of HF Index (CC-SCHFI)	To evaluate the influence of the total mutuality and its dimensions on self-care maintenance, management, and confidence in HF patient–caregiver dyads	- Higher patient mutuality was associated with better self-care maintenance and confidence, and higher caregiver mutuality was associated with better caregiver self-care confidence - Patients and caregivers respond better to symptoms when they experience feelings of appreciation, help, comfort, confidence, and emotional support -If one member of the dyad feels higher mutuality toward the other member of the dyad, this improves only his or her own self-care confidence and not the self-care confidence of the other member of the dyad	The quality of the relationship within the dyad is a protective factor in illness management as mutuality improves self-care in the dyad; self-care maintenance in both patients and caregivers could be improve by shared pleasurable activities within the dyad.
(Dellafiore et al., 2019) [53]	Italy; 366 dyads (patient–spousal and non-spousal caregiver); 56% male patients; M_age_ = 71.9 (patient) and M_age_ = 53.8 (caregiver)	Mutuality Scale; Hospital Anxiety and Depression Scale (HADS)	To evaluate the associations among mutuality, anxiety, and depressionin HF patient–caregiver dyads	- Higher patient mutuality in his/her relationship with the caregiver was associated with lower patient anxiety and depression - Higher patient mutuality was associated with higher caregiver depression	Good relationship with patients is not “protective” against anxiety and depression in caregivers.
(Bugajski et al., 2020) [54]	Italy; 277 dyad; (patient–spousal and non-spousal caregiver); 55% male patients; M_age_ = 75.5 (patient) and M_age_ = 52.8 (caregiver)	Self-care of HF Index (SCHFI) and Caregiver Contribution to Self-care of HF Index (CC-SCHFI); The Dyadic Symptom Management Type (DSMT)	To examine the role of HF self-care dyadic type congruence on patient self-care (maintenance, symptom perception, and management)	- Dyad congruence was a significant predictor of patient’s symptom perception scores but not self-care maintenance or management. - Caregiver’s satisfaction with the dyad was differentially and significantly associated with self-care (inversely with patient self-care maintenance and positively with patient self-care management)	Congruence in HF dyads is associated with better patient symptom perception; the important factor of dyadic HF self-care is the relationship between partners.

## Data Availability

Not applicable.
